# Reliable Semantic Communication System Enabled by Knowledge Graph

**DOI:** 10.3390/e24060846

**Published:** 2022-06-20

**Authors:** Shengteng Jiang, Yueling Liu, Yichi Zhang, Peng Luo, Kuo Cao, Jun Xiong, Haitao Zhao, Jibo Wei

**Affiliations:** College of Electronic Science and Technology, National University of Defense Technology, Changsha 410073, China; jiangshengteng@nudt.edu.cn (S.J.); zhangyichi13@nudt.edu.cn (Y.Z.); pengluo.eric@outlook.com (P.L.); xj8765@nudt.edu.cn (J.X.); haitaozhao@nudt.edu.cn (H.Z.); wjbhw@nudt.edu.cn (J.W.)

**Keywords:** semantic communication, knowledge graph, semantic extraction, semantic restoration

## Abstract

Semantic communication is a promising technology used to overcome the challenges of large bandwidth and power requirements caused by the data explosion. Semantic representation is an important issue in semantic communication. The knowledge graph, powered by deep learning, can improve the accuracy of semantic representation while removing semantic ambiguity. Therefore, we propose a semantic communication system based on the knowledge graph. Specifically, in our system, the transmitted sentences are converted into triplets by using the knowledge graph. Triplets can be viewed as basic semantic symbols for semantic extraction and restoration and can be sorted based on semantic importance. Moreover, the proposed communication system adaptively adjusts the transmitted contents according to channel quality and allocates more transmission resources to important triplets to enhance communication reliability. Simulation results show that the proposed system significantly enhances the reliability of the communication in the low signal-to-noise regime compared to the traditional schemes.

## 1. Introduction

In recent years, wireless communication technology has developed rapidly, bringing great convenience to human life. Fifth-generation (5G) wireless communication technology has played an important role in smart cities, autonomous driving, telemedicine, and other fields [1]. However, with the gradual increase in the communication rate, the explosive growth of data has created enormous challenges for wireless communication technology [2]. According to the forecast from the International Telecommunication Union (ITU), the annual growth rate of the global mobile data stream will reach up to 55% by 2030 [3]. Moreover, the transmission rate of existing communication technologies has gradually approached the Shannon capacity [4], which cannot meet the continuously growing communication demands in the future 6G era. In the future, the 6G communication system will play an important role in remote holography [5], digital twin [6], and other application fields. Therefore, the sixth-generation wireless communication system needs to provide an ultra-high peak rate, ultra-large user experience rate, and ultra-low network latency, which will consume more limited available spectrum and power and bring huge challenges to communication technology. Semantic communication is one of the effective techniques used to overcome these challenges [7].

Semantic communication, as a revolution against traditional communication, is a new communication paradigm [8]. The concept of semantic communication was first proposed by Weaver (1949) [9]. After Shannon (1948) put forward the classical information theory [4], Weaver proposed that communication should be divided into three different layers, namely the technical layer, semantic layer, and effectiveness layer. The technical layer represents traditional communication, focusing on “how to accurately transmit communication symbols”. The semantic layer focuses on “how to accurately convey the meaning of communication symbols”; the effectiveness layer focuses on “how the received meaning effectively affects the receiver’s behavior”. Compared with traditional communication, semantic communication aims to reduce the uncertainty of message understanding between the transmitter and the receiver. Moreover, semantic communication mainly transmits semantic-relevant information, which greatly reduces the amount of redundant data. Therefore, semantic communication is a suitable technology (against the scenarios) with limited communication bandwidth and a low signal-to-noise ratio (SNR) [10,11].

However, some fundamental problems of semantic communication have not been effectively solved. One of them is semantic representation, which limits the development of semantic communication [7]. Regarding semantic representation—existing research studies tend to use transmitted content features to represent the semantics. This representation lacks human language logic and cannot be interactive verification with human understanding [12]. To solve this problem, we considered using the knowledge graph instead of features to represent semantics. The knowledge graph can decompose text into multiple semantic units without losing semantics [13], ensuring the accuracy of semantic representation. The basic structure of the knowledge graph is a triplet in the form of an “entity-relation-entity” [13]. From the linguistic point of view, a single entity may have multiple types of semantic information. The specific semantic information can be determined after a relationship is formed between entities, so the triplet in the knowledge graph can be regarded as the smallest semantic symbol. There have been some research studies exploring the relationship between the knowledge graph and semantics. Jaradeh et al. (2019) proposed that the knowledge graph was the next-generation infrastructure for semantic scholarly knowledge [14]. Mosa (2021) proposed that the knowledge graph could help with semantic category prediction [15]. Zhou et al. (2022) combined the knowledge graph with semantic communication to improve the validity of communication [16]. Thus, the knowledge graph can effectively represent semantics; we investigated the semantic communication system based on the knowledge graph (SCKG) for improving communication reliability. The main contributions of this paper are summarized as follows:

- A semantic extraction method is proposed to extract triplets from transmitted text to represent its core semantic information, reducing the information redundancy of the transmitted text.
- A semantic restoration method based on text generation from the knowledge graph is proposed, which completes the semantic restoration process by reconstructing the text structure between entities and relations.
- A novel semantic communication system was developed, which can sort triplets based on semantic importance and adaptively adjust the transmitted contents according to the channel quality.

The rest of this paper is organized as follows. Section 2 briefly reviews the related work. Section 3 details the proposed system and the semantic extraction and restoration methods used in the model. Experimental results are presented in Section 4 to verify the performance of the proposed model. Finally, Section 5 concludes this paper.

## 2. Related Work

### 2.1. Semantic Communication Development

Due to technical limitations in the early stage of communication development, researchers have focused on solving engineering problems at the technical layer and postponed the study at the semantic layer. However, this does not mean that the research on semantic communication will be shelved. With the advancements in technology, the semantic problem has become an urgent problem that needs to be solved in the communication field [17].

In terms of theoretical research, Carnap et al. (1954) first proposed the concept of the semantic information theory to supplement the classical information theory [18]. They thought that the semantic information contained in the sentence should be defined based on the logical probability of the content of the sentence. Floridi (2004) proposed a theory of strongly semantic information [19] and pointed out the problem that sentence contradictions will have infinite information. Bao et al. (2011) put forward a general model of semantic communication, using a factual statement in the propositional logic form to represent semantics [20]. Moreover, the semantic entropy, semantic noise, and semantic channel capacity were defined in [20]. Based on the literature [20], Basu et al. (2012) provided a detailed explanation of the relationship between semantic entropy and information entropy, and they defined the concepts of semantic ambiguity and semantic redundancy [21]. In [22], Lan et al. (2021) proposed that semantic communication can be divided into human-to-human, human-to-machine, and machine-to-machine sub-areas, which broadened the scope of semantic communication.

On the other hand, the rapid development of neural networks and artificial intelligence technology promotes the progress of technical research in semantic communication. In terms of semantic coding, the authors of [23] proposed a joint source-channel coding for semantic information with a bidirectional long short-term memory model (BILSTM). As an extension of the literature [23], Rao et al. (2018) presented a variable-length joint source-channel coding of semantic information [24]. In [25], Liu et al. (2022) proposed a semantic encoding strategy based on parts-of-speech and context-based decoding strategies, which enhanced communication reliability from the semantic level. Based on the semantic communication framework, Xie et al. (2021) proposed a deep learning-based semantic communication model [26], which used word embedding technology to map text to semantic space and then performed source-channel joint encoding for semantic information by using the transformer framework [27]. Furthermore, the authors of [28] proposed a lightweight distributed semantic communication system for the application scenario of the internet of things (IoT), which reduced the cost of IoT devices. The authors of [29] proposed a semantic communication model based on reinforcement learning to investigate the impact of noisy environments on semantic information. In different information forms, Weng et al. (2021) proposed a semantic communication model for speech transmission [30]. In [31], Hu et al. (2022) proposed a robust end-to-end semantic communication system to combat the semantic noise for image transmission. Moreover, a semantic communication model based on multi-information modalities was developed in [32]. Regarding semantic representation, Zhou et al. (2022) used the transformer for semantic extraction and semantic restoration [33].

### 2.2. Performance Metrics

Semantic communication, different from traditional communication systems, does not emphasize the perfect recovery of the transmitted message, but rather on the receiver correctly understanding the message in the same way as the transmitter. As a result, performance metrics commonly used in traditional communication systems (e.g., bit error rate and symbol error rate) are no longer suitable for semantic communication. Hence, this paper uses the bilingual evaluation understudy (BLEU) score [34], a metric for evaluation of translation with the explicit ordering (METEOR) score [35], and the semantic similarity score [36], as performance metrics.

#### 2.2.1. BLEU Score

BLEU is currently the most commonly used metric in text evaluation [37]. It evaluates the similarity by counting the number of the same n-grams between transmitted and received texts, where n-gram means *n* consecutive words in the text. The formula can be expressed as

(1) $$\log\mathrm{BLEU}=\min\left(1-\frac{l_{\hat{s}}}{l_{s}},0\right)+\sum_{n=1}^{N}\omega_{n}\log p_{n}$$

where *s* and $\hat{s}$ denote the transmitted sentence and restored sentence, respectively. $l_{s}$ and $l_{\hat{s}}$ are the lengths of the transmitted sentences *s* and restored sentence $\hat{s}$, respectively. $\omega_{n}$ represents the weight of n-grams, and $p_{n}$ denotes the precision of n-grams.

#### 2.2.2. METEOR Score

METEOR extends the synonym set by introducing external knowledge sources, such as WordNet [38]. Furthermore, it uses precision $P_{m}$ and recall $R_{m}$ to evaluate the similarity between transmitted and received texts. The formula is given as follows

(2) $$F_{\mathrm{mean}\;}=\frac{P_{m}R_{m}}{\alpha P_{m}+\left(1-\alpha \right)R_{m}}$$

(3) $$\mathrm{METEOR}=\left(1-\mathrm{Pen}\right)F_{\mathrm{mean}}$$

where α is the hyperparameter according to WordNet, $F_{\mathrm{mean}}$ represents the harmonic mean combining $P_{m}$ and $R_{m}$, and Pen is the penalty coefficient.

#### 2.2.3. Semantic Similarity Score

The semantic similarity score converts text into vectors by using the BERT model [39]. It evaluates the semantic similarity between sentences by comparing the degree of similarity between vectors. For the transmitted sentence’s vector v(s) and the received sentence’s vector $v(\hat{s})$, the semantic similarity score can be expressed as

(4) $$sim_{v}\left(s,\hat{s}\right)=\frac{v\left(s\right)\cdot {\left(v\left(\hat{s}\right)\right)}^{T}}{\|v\left(s\right)\|\cdot \|v\left(\hat{s}\right)\|}$$

All the performance metrics introduced above take values between 0 and 1. A higher score given by the performance metrics means that the received text’s semantic is closer to the transmitted text’s semantic; 0 means semantically irrelevant; 1 means semantically consistent.

## 3. System Model

**Figure 1:**
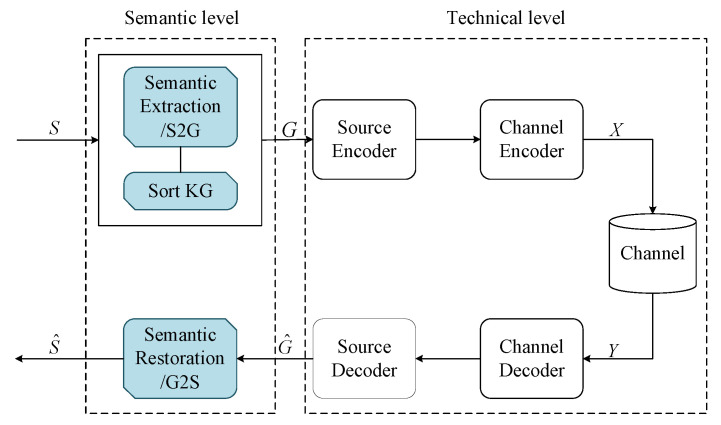
The structure of the proposed semantic communication system based on the knowledge graph, including the semantic extraction module, traditional communication architecture, and semantic restoration module.

As shown in Figure 1, the structure of the proposed system consists of a semantic extraction module, traditional communication architecture, and semantic restoration module. The proposed system can be divided into two levels, which are the semantic level and the technical level. The structure of the technical level is the same as that of the traditional communication system; thus, we mainly introduce the details at the semantic level. At the transmitter, the semantic extraction module can extract the knowledge graph (KG) of the transmitted sentence to represent its semantics. More importantly, the knowledge graph is sorted according to semantic importance. At the receiver, the semantic restoration module can recover the transmitted sentence according to the received knowledge graph.

**Figure 2:**
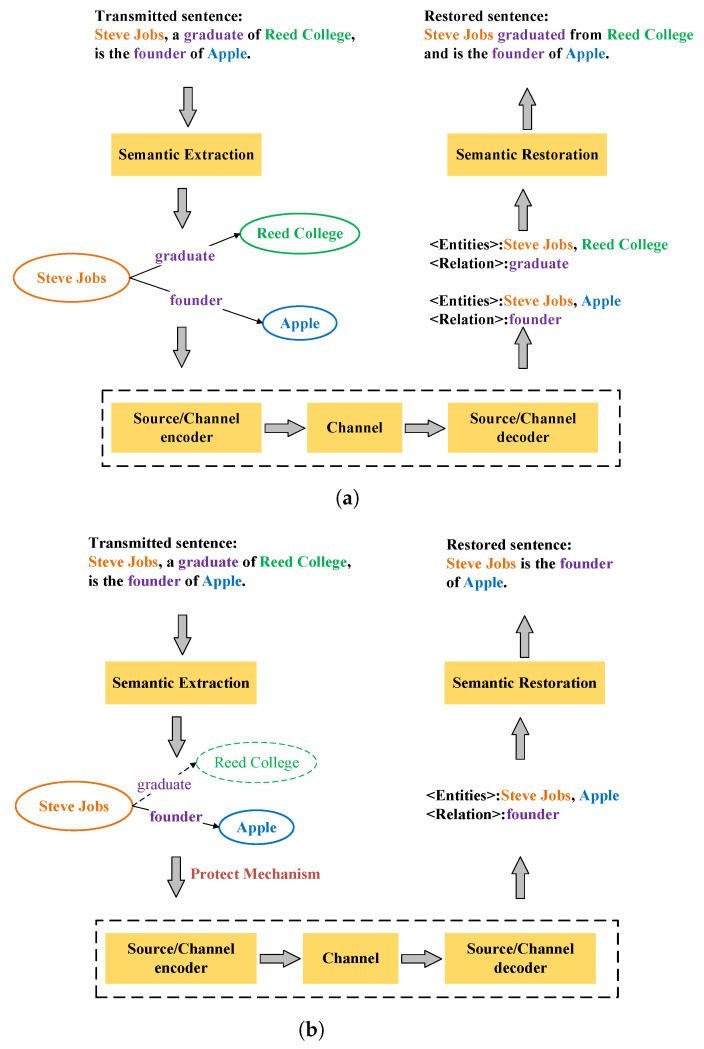
Examples of the proposed semantic communication system in different channel qualities. (**a**) An example of the proposed semantic communication system when the channel quality is good. (**b**) An example of the proposed semantic communication system when the channel quality is poor.

Figure 2 shows examples of the proposed semantic communication system in different channel qualities. At the transmitter, the transmitted sentence is first converted into the knowledge graph through the semantic extraction module. Next, the transmitter adjusts the knowledge graph according to the channel quality. Then, the knowledge graph is transmitted through the channel. With the noisy knowledge graph received, the semantic is recovered through the semantic restoration module. In Figure 2a, when the channel quality is good, the transmitted sentence and the restored sentence convey the same semantics although they have different sentence structures. When the channel quality is poor, all triplets cannot be transmitted correctly. Therefore, the proposed semantic communication system chooses to transmit the most important triplet. When it comes to Steve Jobs, people tend to care about his relationship with Apple rather than the college he graduated from. As shown in Figure 2b, the transmitter only sends “< Steve Jobs-founder-Apple” when the channel quality is poor.

### 3.1. Semantic Extraction Method

**Figure 3:**
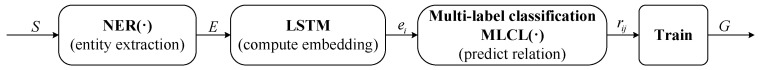
The deep learning network structure for the semantic extraction method.

To represent the semantic information correctly, the semantic extraction module at the transmitter uses a deep learning network to extract the knowledge graph from the transmitted sentence. Let $S2G_{\theta }\left(•\right)$ be the function of the proposed semantic extraction method, which takes the sentence $S=\left[w_{1},w_{2},\cdots ,w_{m}\right]$ as input and its corresponding output is the knowledge graph *G*, where $w_{m}$ is the *m*th word in the sentence. The deep learning network structure for the semantic extraction method is shown in Figure 3.

In particular, we used the pipeline method to extract the knowledge graph, which means extracting the entities in *S* and then predicting the relations between entities. Firstly, we used a well-established named entity recognition model (NER) to extract the entities [40]. This model is based on the conditional random field classifier and Gibbs sampling. The conditional random field classifier combines the characteristics of the maximum entropy model and the hidden Markov model, and it is often used to deal with sequence labeling tasks, such as parts-of-speech tagging and named entity recognition. Gibbs sampling is a method of generating Markov chains that can be used for Monte Carlo simulations. Based on the conditional random field classifier and Gibbs sampling, NER is trained by using a large amount of manually annotated text and can recognize entities from given sentences. Therefore, the entities in the transmitted sentence can be expressed as

(5) $$E=\left[en_{1},en_{2},\cdots ,en_{i},\ldots ,en_{L}\right]=\mathrm{NER}\left(S\right)$$

where $en_{i}$ represents the *i*th entity in the sentence, *L* is the total number of entities contained in the sentence.

After extracting entities from *S*, we predict the relations between the two entities. Firstly, the embedding of each word $w_{j}$ in the entity $en_{i}$ is averaged to obtain the entity’s embedding. The embedding of $w_{j}$ can be obtained by using a long short-term memory model (LSTM) [41] to encode $w_{j}$ and its context. The formula is given as follows

(6) $$\mathrm{emb}\left(w_{j}\right)=\mathrm{LSTM}\_\mathrm{encode}\left(w_{j},w_{<j},w_{>j}\right)$$

Therefore, the *i*th entity’s embedding $e_{i}$ can be represented as

(7) $$e_{i}=\frac{1}{\mathrm{Len}\left(en_{i}\right)}\sum_{w_{j}\in en_{i}}\mathrm{emb}\left(w_{j}\right)$$

where $\mathrm{Len}\left(en_{i}\right)$ is the number of words in the entity $en_{i}$.

Then we feed the entity embeddings into a multi-label classification layer MLCL(•) to predict the relations. The multi-label classification layer MLCL(•) can take in two entities and predict the possible relation set. To prevent these two entities from being irrelevant, the relation set includes the “no-relation” type. The relation set between the *i*th entity and the *j*th entity can be represented as

(8) $$r_{ij}=\mathrm{MLCL}\left(e_{i},e_{j}\right)$$

Since the knowledge graph is made of entities and relations, the probability of extracting a graph from a given sentence is equivalent to the product of the probability of extracting the relation set given any two entities. The formula can be expressed as

(9) $$p\left(G|S\right)=\prod_{i=0}^{L}\prod_{j=0}^{L}p\left(r_{ij}|e_{i},e_{j},S\right)$$

Based on the probability p(G∣S), we can denote the loss function of the proposed semantic extraction method by using the negative log-likelihood loss, which can be formulated as

(10) $$\begin{array}{cc} L_{S2G}\left(\theta \right) & =E[-\log p(G|S;\theta )]\\ \; & =E\left[-\log\prod_{i=0}^{L}\prod_{j=0}^{L}p\left(r_{ij}|e_{i},e_{j},S;\theta \right)\right] \end{array}$$

where θ is the network parameter set of the deep learning network, which is shown in Figure 3.

Utilizing the loss function $L_{S2G}$, the optimal parameter set $\theta^{*}$ can be easily found using the gradient descent method. Consequently, the details of the proposed semantic extraction method can be summarized in Algorithm 1.
**Algorithm 1** The proposed semantic extraction method**Input:** the transmitted sentence *S*1:Build entity set *E* by Equation (5)2:**for** each $en_{i}\in E$ **do**3:   Compute the embedding $e_{i}$ by Equations (6) and (7)4:**end for**5:Construct the relation set according to Equation (8)6:Compute loss function $L_{S2G}\left(\theta \right)$ according to Equation (10)7:Train $\theta \to \theta^{*}$**Output:** The knowledge graph *G*

### 3.2. Semantic Restoration Method

**Figure 4:**
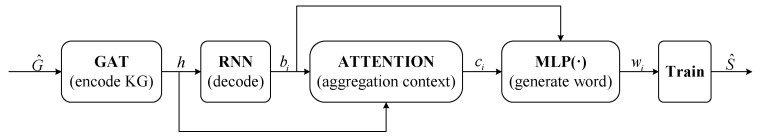
The deep learning network structure for the semantic restoration method.

The proposed semantic restoration method—similar to the proposed semantic extraction method—uses deep learning to generate sentences from the received knowledge graph. The generated sentence can help the receiver understand the semantics of the transmitted sentence. Let $G2S_{\varphi }\left(•\right)$ be the function of the proposed semantic restoration. The input of $G2S_{\varphi }\left(•\right)$ is the received knowledge graph $\hat{G}$ and its output is the restored sentence $\hat{S}$. The deep learning network structure for the semantic restoration method is shown in Figure 4.

At first, we encoded the received knowledge graph $\hat{G}$ to convert it to the embedding, which could be processed by the deep learning network. Specifically, we used the graph attention network (GAT) [42] to calculate the embedding of the received knowledge graph $\hat{G}$. GAT is a representative graph convolutional network that can encode the knowledge graph by introducing the attention mechanism into the knowledge graph. Therefore, the embedding of $\hat{G}$ can be represented as

(11) $$h=\mathrm{GAT}(\hat{G})$$

After obtaining the embedding *h*, we used the recurrent neural network (RNN) and the attention mechanism to generate the sentence word by word. Each step of RNN can produce a word embedding. In the *i*th step, the embedding $b_{i}$ can be represented as

(12) $$b_{i}=\mathrm{RNN}\left(b_{i-1},w_{i-1}\right)$$

where $w_{i-1}$ is the i−1th word in the generated sentence, $b_{i-1}$ is the embedding produced in the i−1th step. To improve the accuracy of the generated sentence, the attention mechanism was used to obtain the embedding of contextual information. The formula can be described as

(13) $$c_{i}=\mathrm{ATTENTION}\left(b_{i},h\right)$$

where $c_{i}$ denotes the contextual information of the *i*th word. Then we fed the word embedding $b_{i}$ and the contextual information $c_{i}$ into a multilayer perceptron (MLP) to generate the *i*th word $w_{i}$.

Consequently, the generation of $w_{i}$ based on the received knowledge graph $\hat{G}$ and all previously generated words $w_{<i}$ was fulfilled by predicting the word $w_{i}$ through MLP with the assistance of the word embedding $b_{i}$ and the contextual information $c_{i}$. Thus, the probability of recovering word $w_{i}$ can be represented as

(14) $$p\left(w_{i}|w_{<i},\hat{G}\right)\propto \exp\left(\mathrm{MLP}\left(\left[b_{i};c_{i}\right]\right)\right)$$

In summary, the probability of generating a sentence from the received knowledge graph $\hat{G}$ is equivalent to the product of the probability of generating each word. The probability can be described as

(15) $$p\left(\hat{S}|\hat{G}\right)=\prod p(w_{i}|w_{<i},\hat{G})$$

Similarly, we used the negative log-likelihood loss to denote the loss function of the proposed semantic restoration method according to the probability $p(\hat{S}|\hat{G})$. The loss function can be represented as

(16) $$\begin{array}{cc} L_{G2S}\left(\varphi \right) & =E[-\log p\left(\hat{S}|\hat{G};\varphi \right)]\\ \; & =E[-\log\prod p(w_{i}|w_{<i},\hat{G};\varphi )] \end{array}$$

where φ is the network parameter set of the deep learning network, which is shown in Figure 4. Finally, the gradient descent can be used to find the optimal parameter set $\varphi^{*}$ for minimizing the loss function $L_{G2S}\left(\varphi \right)$.

The details of the proposed semantic restoration process are summarized in Algorithm 2.
**Algorithm 2** The proposed semantic restoration method**Input:** the received knowledge graph $\hat{G}$1:Compute the embedding of $\hat{G}$ by Equation (11)2:**while**$w_{i}$ is not the satisfied end feature **do**3:   Compute $b_{i}$ by Equation (12)4:   Compute the contextual information $c_{i}$ by Equation (13)5:   Generate word $w_{i}$ according to Equation (14)6:**end while**7:Compute the loss function $L_{G2S}\left(\varphi \right)$ according to Equation (16)8:Train $\varphi \to \varphi^{*}$**Output:** the knowledge graph $\hat{S}$


### 3.3. System Process

**Figure 5:**
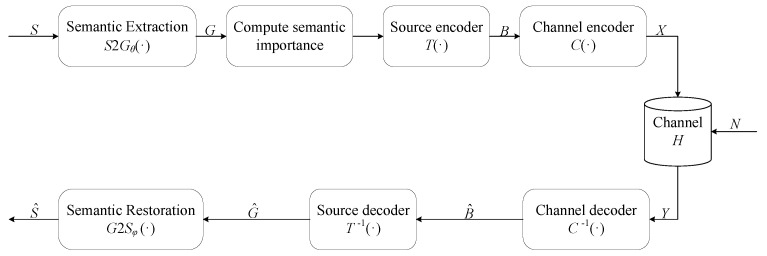
The overall process of the proposed semantic communication system based on the knowledge graph, combining the proposed semantic extraction method, the proposed semantic restoration method, and the traditional communication architecture.

In this section, we introduce the overall process of the proposed semantic communication system. Let $S=\left[w_{1},w_{2},\cdots ,w_{m}\right]$ be the transmitted sentence, where $w_{m}$ is the *m*th word in the sentence. As shown in Figure 5, with the help of the proposed semantic extraction method ${S2G}_{\theta }\left(•\right)$, the transmitter converts the transmitted sentence *S* to the knowledge graph *G*, which can be represented as $G={S2G}_{\theta }\left(S\right)$. The knowledge graph *G* consists of *n* triplets and it can be formulated as $G=\left[g_{1},g_{2},\cdots ,g_{n}\right]$.

Using the proposed semantic extraction method, the transmitted sentence is converted into a series of triplets. In this process, the semantics of the transmitted sentence are extracted without losing semantics [13]. During transmission, these triplets are independent of each other, which means that errors in some triplets will not affect other triplets. However, in Markov models, once there is a transmission error, the whole transmitted sentence will be affected. Therefore, the proposed semantic communication system is more robust under a low SNR. Moreover, different semantic basic symbols (triplets) have semantic importance in semantic communication, unlike bits or symbols that are treated equally in traditional communication, such as longer-range models and Markov chain-based probabilistic models. These triplets (with semantic importance) should be treated differently. The triplets with important semantics should be allocated with many time slots and bandwidth resources. When the channel quality is extremely poor, instead of transmitting all triplets, which cannot be guaranteed by the channel, it is better to ensure that the most important triplet can be transmitted correctly. When the channel quality is better, the system can adjust the sending content according to semantic importance. Motivated by the different triplets with semantic importance, we sort these triplets according to their semantic similarity scores:

(17) $$sim_{v}\left(s,g_{i}\right)=\frac{v\left(s\right)\cdot {\left(v\left(g_{i}\right)\right)}^{T}}{\|v\left(s\right)\|\cdot \|v\left(g_{i}\right)\|}$$

where $g_{i}$ denotes the *i*th triplet in *G*. Table 1 shows an example of semantic importance. From Table 1, “< Steve Jobs – founder-Apple>” is more important than “< Steve Jobs – graduate-Reed College >”, which is also in line with human perception.

Based on the sorted triplets, we can adaptively adjust the number of transmitted triplets according to the channel quality. When the channel quality is extremely poor, we only transmit the most significant triplet and use the communication resources of triplets not transmitted to protect it. As the channel quality improves, we increase the number of transmitted triplets.

After the transmitted knowledge graph *G* is obtained, the transmitter first maps it into a binary bit stream $B=T\left(G\right)$, and then feeds the binary bit stream into the channel encoder to cope with the effects of channel noise and distortion. Therefore, the whole process of the transmitter can be represented as

(18) $$X=C\left(T\left(G\right)\right)$$

where T(•) and C(•) denote the source encoder and the channel encoder, respectively. If *X* is sent, the received signal can be represented as

(19) Y=HX+N

where *H* is the channel coefficient and $N∼CN\left(0,\sigma_{n}^{2}\right)$ denotes the additive white Gaussian noise.

After obtaining the received signal, the receiver will decode it to recover the transmitted knowledge graph. Defining $C^{-1}\left(•\right)$ and $T^{-1}\left(•\right)$ as the channel decoder and the source decoder, respectively, the received knowledge graph $\hat{G}$ can be represented as

(20) $$\hat{G}=T^{-1}(C^{-1}\left(Y\right))$$

Then we use the proposed semantic restoration method $G2S_{\varphi }\left(•\right)$ to obtain the restored sentence $\hat{S}$.

(21) $$\hat{S}=G2S_{\varphi }\left(\hat{G}\right)$$

The process of the proposed semantic communication system is shown in Algorithm 3.
**Algorithm 3** Process of the proposed semantic communication system.**Input:** The transmitted sentence *S*1:**Transmitter:**2:   Extract the knowledge graph by Algorithm 13:   **for** i=1 to *n* **do**4:      Compute the semantic importance of $g_{i}$ by Equation (17)5:   **end for**6:   Sort the knowledge graph according to the semantic importance7:   Adjust the number of transmitted triplets according to the channel quality8:   $C\left(T\left(G\right)\right)\to X$9:   Transmit *X* over the channel10:**Receiver:**11:   Receive *Y*12:   $T^{-1}\left(C^{-1}\left(Y\right)\right)\to \hat{G}$13:   Restore the sentence $\hat{S}$ by Algorithm 2**Output:** The restored sentence $\hat{S}$


## 4. Experimental Results

In this section, we compare the proposed SCKG with other traditional models under different channels, including the AWGN channel and the Rayleigh fading channel to verify the effectiveness of SCKG. In Table 2, we introduce the models used in the experiments, including their general features and technical methods. It is worth noting that the traditional communication models are not the only ones mentioned in Table 2. The source coding can also choose arithmetic coding, L–Z coding, and other coding methods. Identically, the channel coding can also choose turbo code, polar code, and other coding methods.

### 4.1. Experimental Settings

In the simulation, the adopted dataset was the WebNLG dataset [45], which is usually used to generate sentences from knowledge graphs. Each data in the dataset consists of multiple triplets and their corresponding sentences. After preprocessing the dataset, we obtained 12,597 training data, 1746 validation data, and 2493 test data. The training and testing environment was Ubuntu16.04+CUDA10.1, the selected deep learning framework was PyTorch 1.6.0. The training settings of the semantic extraction method and the semantic restoration method are shown in Table 3.

In the experiment, the test data of WebNLG were transmitted sentence-by-sentence to the transmitter. Then we obtained the restored sentences by using the above-mentioned methods at the receiver. After the restored sentences were obtained, the experimental results could be calculated according to the performance metrics.

For the benchmark, we adopted the traditional communication architecture with source coding and channel coding, where source coding could use Huffman coding, arithmetic coding, L–Z coding, etc., and channel coding could use LDPC coding, turbo code, polar code, etc. For simplicity, we adopted the combination of Huffman coding and LDPC coding (named “Huffman + LDPC”). Moreover, we considered another two methods as ablation experiments to validate the effectiveness of the proposed model. One involved using the proposed model without adaptive transmission and semantic restoration (named the “Proposed model without AT and SR”), and the other involved using the proposed model without adaptive transmission (named the “Proposed model without AT”).

### 4.2. Experimental Result Analysis

#### 4.2.1. Performance of the Proposed Semantic Communication System

**Figure 6:**
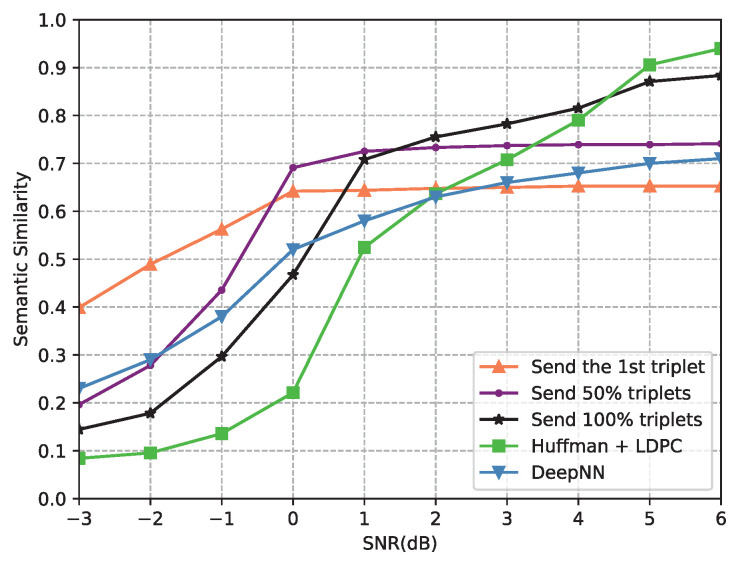
Semantic similarity versus the SNR under the AWGN channel, with send the 1st triplet; send 50% triplets; send 100% triplets; Huffman + LDPC; DeepNN.

First, we investigated the effects of the number of triplets on the semantic performance under different SNRs. Here, we considered three strategies, one strategy was to send the first triplet (named “Send the 1st triplet”), and the other two schemes involved sending 50% triplets (named “Send 50% triplets”) and 100% triplets (named “Send 100% triplets”), respectively. Moreover, we compared these three strategies with the benchmark and an end-to-end deep learning-based communication system proposed in [23] (named DeepNN). Figure 6 shows the performance of the semantic similarity versus the SNR in this experiment. From Figure 6, “Send the 1st triplet” has the best semantic similarity under a low SNR because it uses the most resources to protect the first triplet. With the SNR becoming better, “Send 50% triplets” has better performance because “Send the 1st triplet” transmits limited semantics, and the accuracy of the scheme “Send 100% triplets” cannot be guaranteed due to the channel distortion. The semantic similarity of “Send 100% triplets” is above the others at a high SNR, which is reasonable due to the error-free transmission when the channel quality is good. Meanwhile, all three strategies outperformed the benchmark and DeepNN in their superior SNR regions. According to Figure 6, it is reasonable to send the most important triplet in the low SNR region, send 50% triplets in the medium SNR region, and send 100% triplets in the high SNR region.

**Figure 7:**
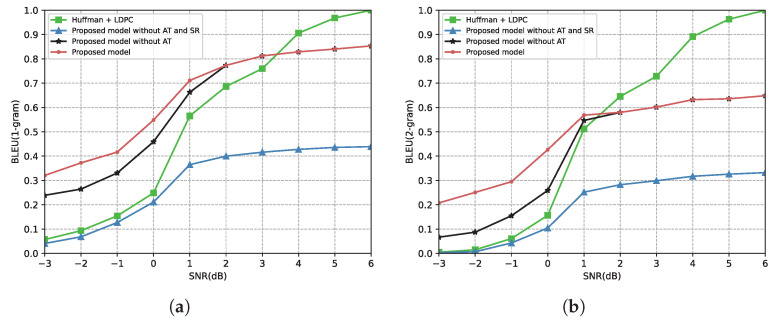
BLEU score versus the SNR over the AWGN channel. (**a**) BLEU(1-gram) score over the AWGN channel. (**b**) BLEU(2-gram) score over the AWGN channel.

Figure 7 demonstrates the relationship between the SNR and the BLEU score under the AWGN channel. From Figure 7, the proposed model performs better under a low SNR in terms of the 1-gram BLEU score or 2-gram BLEU score due to the protection of important triplets. Moreover, after converting the received triplets into sentences by using the proposed semantic restoration method, “Proposed model without AT” outperforms “Proposed model without AT and SR” for all SNR regimes. However, the performance of the proposed model is inferior to the traditional communication system in the high SNR region in Figure 7. This is because the proposed semantic restoration method attempts to recover the same semantic rather than the same sentence structure. For example, the transmitted sentence is “Steve Jobs was the founder of Apple”, and the restored sentence is “Steve Jobs founded Apple”. Although the two sentences are semantically consistent, the BLEU score of the proposed scheme is poor.

**Figure 8:**
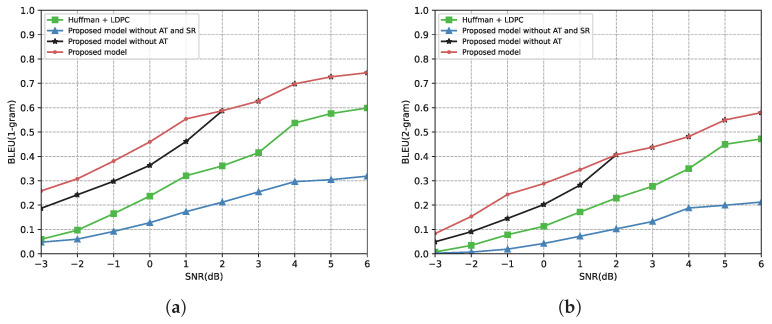
BLEU score versus the SNR over the Rayleigh fading channel. (**a**) BLEU(1-gram) score over the Rayleigh fading channel. (**b**) BLEU(2-gram) score over the Rayleigh fading channel.

Figure 8 shows the relationship between the SNR and the BLEU score under the Rayleigh fading channel. All scores in Figure 8 are lower than the scores in Figure 7 because of the severe impacts of Rayleigh fading. However, the proposed model significantly improves performance compared to the benchmark. From Figure 8, the proposed model outperforms the benchmark across the SNR range over the Rayleigh fading channel, either the 1-gram BLEU score or the 2-gram BLEU score. It reflects that our proposed model is more robust to complex communication environments. Meanwhile, since “Proposed model without AT” and “Send 100% triplets” are identical in the high SNR region, the results of the proposed model and “Proposed model without AT” are the same when the SNR is higher than 2 dB.

**Figure 9:**
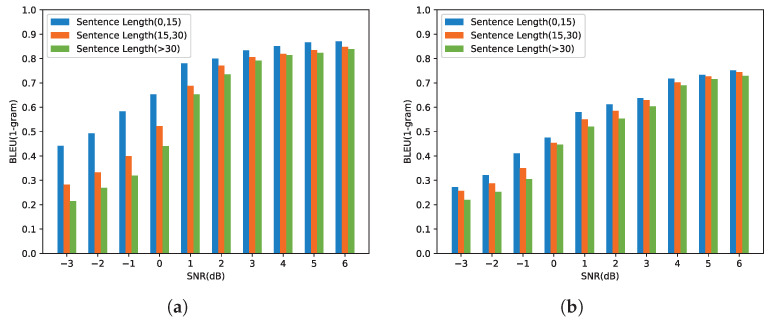
BLEU (1-gram) score versus the SNR with sentence length (0, 15). Sentence Length (15, 30); sentence length (>30). (**a**) BLEU (1-gram) score over the AWGN channel. (**b**) BLEU (1-gram) score over the Rayleigh fading channel.

Since BLEU is an evaluation metric that calculates scores based on word matching, sentence sizes can affect the performance of our proposed model. To research this, we divided the transmitted sentences into three groups—sentence length between 0 and 15, sentence length between 15 and 30, and sentence length greater than 30. Figure 9 shows the relationship between the SNR and the (1-gram) BLEU score under the AWGN channel and the Rayleigh fading channel, respectively. From Figure 9a, “Sentence Length (0, 15)” is significantly higher than the other two groups. This is because the proposed model only transmits the most important triplet in the low SNR, and the length of the restored sentence is limited. In the low SNR region, the BLEU score decreases as the sentence length increases. With the SNR increasing, the number of the transmitted triplets increases, and the gaps between the different groups narrow. In Figure 9b, the gaps between the different groups are not obvious due to the effects of Rayleigh fading.

**Figure 10:**
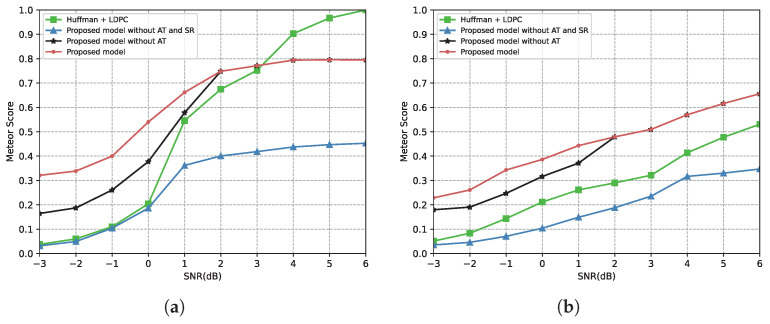
(**a**) METEOR score versus the SNR over the AWGN channel. (**b**) METEOR score versus the SNR over the Rayleigh fading channel.

Figure 10 shows the METEOR score versus the SNR over the AWGN channel and the Rayleigh fading channel. From Figure 10a, the score of the benchmark is close to 1 and higher than our proposed model when the SNR is above 4 dB. This is because the few errors that occurred during the transmission were corrected by the channel coding at a high SNR; the benchmark could restore the transmitted sentence without distortion. However, our proposed model discards the information of sentence structure during transmission. When the SNR is less than 4 dB, the channel coding cannot correct all errors during transmission. In this situation, the METEOR score of the benchmark degrades rapidly. However, the proposed model reduces the number of transmitted triplets and protects important triplets in this case, which leads to a better performance in the low SNR region. From Figure 10b, even under the Rayleigh fading channel, our model outperforms the benchmark in all SNR regions.

**Figure 11:**
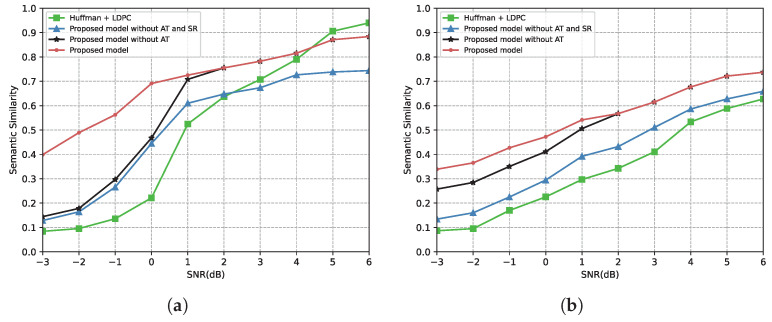
(**a**) Semantic similarity versus the SNR over the AWGN channel. (**b**) Semantic similarity versus the SNR over the Rayleigh fading channel.

Figure 11 draws the relationship between the SNR and the semantic similarity under the AWGN channel and the Rayleigh fading channel. From Figure 11, the “Proposed model without AT and SR” outperforms the benchmark in the low SNR region under the AWGN channel, while it outperforms the benchmark in all SNR regions under the Rayleigh fading channel. This is because our proposed model splits the transmitted sentence into multiple independent triplets, leading to that, the wrongly transmitted triplets will not affect the semantics of other triplets. However, the benchmark model transmits the sentence as a whole, and if errors occur in the transmission, then the semantics of the sentence are affected. Therefore, when the channel quality is poor, our proposed model can preserve partially correct semantics. Meanwhile, since the semantic similarity based on the BERT model can capture semantic relationships among words, the proposed scheme obtains a higher similarity compared with the BLEU score and METEOR score.

To ensure the fairness of the comparison of experimental results, we computed the time complexities of all strategies. We transmitted 1000 sentences from the transmitter to the receiver by using different strategies and calculated the average execution time. All tests were run on Python and were performed by the computer with AMD Ryzen 7 4800H and NVIDIA GeForce GTX 3060. The results are shown in Table 4. From Table 4, our proposed model increases the computational complexity and improves communication reliability.

#### 4.2.2. Comparison with Other Semantic Communication Models

**Figure 12:**
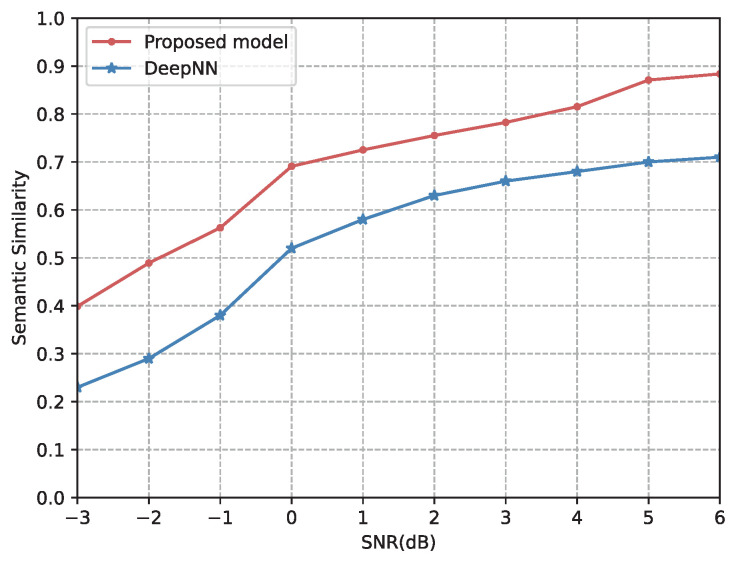
Semantic similarity of our proposed model and DeepNN versus the SNR over the AWGN channel.

To validate that our proposed model is more competitive than existing research, we compared it with the scheme from [23], which adopts an end-to-end deep learning-based communication system for text transmission (named DeepNN). Figure 12 shows the relationship between the SNR and the semantic similarity performance over the AWGN channel. From Figure 12, our proposed model outperforms DeepNN across the entire SNR region. The reasons are two-fold. First, by using triplets as semantic basic symbols, our proposed model can extract lossless semantics. Moreover, the important triplets are allocated more transmission resources in our proposed model, which effectively protects the importance of the semantics. However, DeepNN uses a fixed bit length to encode sentences of different lengths, resulting in a partial loss of semantics.

## 5. Conclusions

In this paper, the reliable semantic communication assisted by the knowledge graph was studied, which overcomes the problem that the meaning of data represented by the features of the deep learning model cannot be explainable [26,28]. Specifically, we proposed a semantic extraction scheme that transforms the transmitted sentence into multiple triplets with semantic importance. Moreover, an adaptive transmission scheme is proposed, in which the important triplets are allocated more communication resources to combat channel distortion. Moreover, a semantic restoration scheme was designed to reconstruct the sentence and recover the whole semantic at the receiver. The simulation results show that the proposed system outperforms the traditional schemes in improving communication reliability, especially in the low SNR regime. However, the optimal number of triplets transmitted over a specific channel is still an ’open question’. In the future, more work is needed to analyze the relationship between the number of triplets and the channel quality.

## Figures and Tables

**Table 1:** An example of semantic importance.

Sentence	Triplets of Knowledge Graph	Semantic Similarity
Steve Jobs, a graduate of Reed	Steve Jobs – graduate-Reed College	0.56
College, is the founder of Apple	Steve Jobs – founder-Apple	0.73

**Table 2:** Introduction to the proposed model and other traditional models.

Model	General Features	Technical Methods
SCKG	(1) Adding the semantic extraction module and semantic restoration module into traditional communication architecture. (2) Using triplets as semantic basic symbols for semantic extraction and restoration.	(1) Semantic extraction—network structure using NER + LSTM. (2) Semantic restoration—network structure using GAT + RNN + ATTENTION.
Huffman [43] + LDPC [44]	(1) Using the traditional communication architecture from Shannon’s information theory. (2) Using Huffman coding as source coding and using LDPC coding as channel coding.	(1) Convert transmitted sentences to bit sequences by using Huffman coding. (2) Using LDPC coding to combat channel distortion.
DeepNN [23]	(1) Using the deep neural network for source-channel joint coding. (2) Replacing source encoding and channel encoding with the encoder of the deep neural network. (3) Replacing source decoding and channel decoding with the decoder of the deep neural network.	(1) Encoder—network structure using BILSTM. (2) Decoder—network structure using LSTM.

**Table 3:** Training settings for semantic extraction and restoration method.

Type	Parameters for Semantic Extraction Method	Parameters for Semantic Restoration Method
Epochs	50	50
Batch size	32	32
Optimizer	Adam	Adam
Learning rate	$5\times 10^{-5}$	$2\times 10^{-4}$
Drop	0	0.1

**Table 4:** The time complexity of all strategies.

Strategies	Time Complexity/s
Huffman + LDPC	2.7324
Proposed model without AT and SR	3.1638
Proposed model without AT	3.7742
Proposed model	3.8539

## Data Availability

Data are available from the authors, on request.
